# Supplementary material for: Development of a Benzophenone-Free Red Propolis Extract and Evaluation of Its Efficacy against Colon Carcinogenesis
Source: Pharmaceuticals (Basel). 2024 Oct 8;17(10):1340. doi: 10.3390/ph17101340 (PMC11510570; doi:10.3390/ph17101340)
Supplement: Supplementary file 1 [file pharmaceuticals-17-01340-s001.zip › pharmaceuticals-3192688-supplementary.pdf]

Supplementary material

Development of a benzophenones-free red propolis extract and evaluation of its efficacy against colon carcinogenesis

Iara Silva Squarisi[1], Victor Pena Ribeiro[1], Arthur Barcelos Ribeiro[1], Letícia Teixeira Marcos de Souza[1], Marcela de Melo Junqueira[1], Kátia Mara de Oliveira[1], Gaelle Hayot[3], Thomas Dickmeis[3], Jairo Kenupp Bastos[2], Rodrigo Cassio Sola Veneziani[1], Sérgio Ricardo Ambrósio[1*], Denise Crispim Tavares[1*].

Affiliation list:

[1]Research Group on Natural Products, Center for Research in Sciences and Technology, University of Franca, Franca, SP, Brazil

[2]School of Pharmaceutical Sciences of Ribeirão Preto, University of São Paulo, Ribeirão Preto, SP, Brazil.

[3]Institute of Biological and Chemical Systems - Biological Information Processing - Karlsruhe Institute of Technology, Karlsruhe, BW, Germany

***Corresponding author:**

SR Ambrósio (sergio.ambrosio@unifran.edu.br) and DC Tavares (denisecrispim2001@yahoo.com), University of Franca, Av. Dr. Armando Salles Oliveira, 201, Franca, 14.404-600, São Paulo, Brazil.

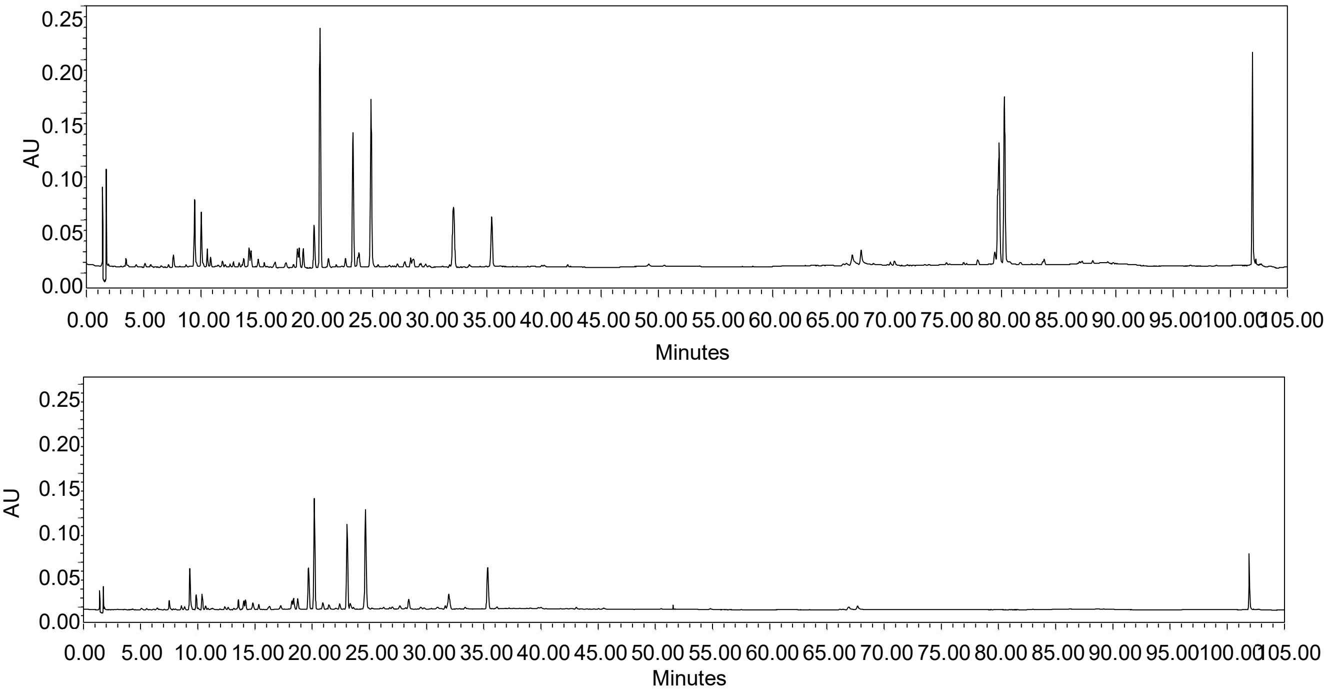

Figure S1. HPL-DAD chromatogram of (A) hydroalcoholic extract of red propolis and (B) benzophenones-free red propolis extract in a method developed by Aldana-Mejía et al. (2021).
